# Interleukin 10 and Tumor Necrosis Factor-Alpha in Pregnancy: Aspects of Interest in Clinical Obstetrics

**DOI:** 10.5402/2012/230742

**Published:** 2012-02-20

**Authors:** Jusciele Brogin Moreli, Ana Maria Cirino Ruocco, Joice Monaliza Vernini, Marilza Vieira Cunha Rudge, Iracema Mattos Paranhos Calderon

**Affiliations:** Post-Graduate Program in Gynecology, Obstetrics and Mastology, Botucatu Medical School, (UNESP) São Paulo State University, 18618-000 Botucatu, SP, Brazil

## Abstract

The purpose of this study was to review the literature regarding the action of the cytokines interleukin 10 (IL-10) and tumor necrosis factor-alpha (TNF-*α*) in pregnancy and to emphasize the factors that are of interest to clinical obstetrics. The literature highlights several actions of IL-10 and TNF-*α* during pregnancy. The actions of these cytokines seem to be antagonistic and dependent on the balance between them, which is orchestrated by the specific immunosuppressive action of IL-10. TNF-*α* has a characteristic inflammatory action, and it is an additional diabetogenic factor in pregnancy. The loss of the control of the production of these cytokines, with increase of TNF-*α*, is related to the risk for developing obstetric complications, particularly recurrent fetal loss, gestational diabetes mellitus, hypertensive syndromes, and fetal growth restriction. However, study results are controversial and are not clearly defined. These issues are attributed to the heterogeneity of the studies, particularly regarding their sample sizes and sources, the evaluation methods, and the multiplicity of factors and conditions that influence cytokine production. These questions are fundamental and should be addressed in future investigations to obtain more consistent results that can be applied to obstetric practice.

## 1. Introduction

Cytokines belong to a diverse family of small, soluble proteins that are expressed by several cell types and tissues and act as immunologic mediators. The subpopulations of these cytokine-producing cells, which classically include T helper 1 (Th1) and T helper 2 (Th2) cells, play fundamental roles in differentiating the nature of the immunologic response. If Th1 cytokines predominate, the immune system will generate a cell-mediated response (cytotoxic type) that is targeted at intracellular pathogens or cancer cells [1]. The Th2 subpopulation induces the production of antibodies against pathogens and thus characterizes the humoral-type immunologic response [2].

To date, at least 22 interleukin types have been identified [3]. The Th1 and Th2 subpopulations were the first classes to be described, generally as with opposing action forms, and they have been thoroughly studied [4]. In humans, the Th1 subpopulation is classically represented by interferon gamma (IFN-*γ*) and by interleukin 12 (IL-12). The Th2 subpopulation is characterized by IL-4, IL-5, and IL-13 [5, 6]. Tumor necrosis factor-alpha (TNF-*α*) and the interleukins 1-beta (IL-1*β*) and IL-6 have clear proinflammatory actions and are classified in the subpopulation of Th1 lymphocytes [6, 7].

Interleukin 10** (**IL-10) is a special type of cytokine that, in humans, plays a dual immunologic role that is either stimulatory and counterregulatory or immunosuppressive. This role excludes it from the Th1 and Th2 classes. However, IL-10 was originally described as a Th2 cytokine because of its anti-inflammatory action in rodents [6, 8, 9].

TNF-*α* is essential in the orchestration of the cytokine cascade, and it is a therapeutic target in many inflammatory diseases. The increased production of TNF-*α* has been related to the pathogenesis of various diseases, including rheumatoid arthritis, Crohn's disease, atherosclerosis psoriasis, sepsis, diabetes mellitus, and obesity [10].

In pregnancy, the immunologic system plays an important role that ensures normal pregnancy development and to promote the development of complications [11]. Pregnancy success appears to rely on a discrete balance between the cytokines Th1 and Th2, which are involved in fetal growth and development [12]. TNF-*α*, IL-1*β*, and IL-6 are some of the fundamental cytokines in early pregnancy. It participates in blastocyst implantation and, adversely, in first-trimester losses. As pregnancy develops, high TNF-*α* concentrations have been related to the development of preeclampsia and gestational diabetes mellitus (GDM), reduced IL-10 levels, and preterm birth [6, 7, 13, 14]. Placental mechanisms are also involved in the regulation of the immunologic response in pregnancy [15]. Trophoblastic and Hofbauer cells in the placenta produce various cytokine types that initially have autocrine and paracrine functions, but they may cause metabolic alterations following the formation of placental villi if they reach maternal circulation [16]. According to the literature, the placenta may contribute to the reduction of maternal immunologic responses and the loss of its regulatory role can result in development of certain complications, such as diabetes mellitus and preeclampsia [14, 16, 17].

Because of the association between immunologic responses and obstetric outcomes, the early identification of immunologic alterations would facilitate the prevention of adverse pregnancy outcomes. However, the immunologic profile of human pregnancy needs to be more clearly defined [6].

From the 1980s to the 1990s, maternal tolerance to alloantigens was explained by the predominance of Th2 immunity over Th1 immunity, which protected the fetus from maternal “attack”. Th1 predominance was observed in cases of recurrent [18, 19] and spontaneous [18, 20] miscarriages and preeclampsia [18, 21]. However, the predominance of Th2 immunity was also reported in cases of recurrent miscarriages [18, 22]; therefore, the Th1/Th2 paradigm is also insufficient to explain the mechanism that prevents the rejection of the fetal allograft. Considering this scenario, some authors have begun to expand this classic paradigm by including responses that are characterized as Th17 and T-regulating (Treg) [18, 23]. Cytokine IL-17 has proinflammatory activity and characterizes the type-Th17 response [23, 24]; it has been described to induce inflammation in autoimmune diseases and acute graft rejection. In contrast, Treg cells induce immunoregulation and tolerance by inhibiting cytokine production and proliferation in T CD4^+^and CD8^+^ cells, immunoglobulin production by B cells, the cytotoxic activity of natural-killer cells, and the maturation of dendritic cells, which results in tolerance induction [18, 25, 26]. 

Several paradigms have been proposed to explain the modulation of the immunologic system in pregnancy [20]. More recently, Treg cells and type-3 T-helper response (Th3) cells have been identified [27, 28] as a result of their immunosuppressive action in pregnancy [29]. IL-10 appears to play a fundamental role among the active mechanisms that foster immunologic modulation in pregnancy [30, 31].

Thaxton and Sharma [32] proposed a contemporary model that explains the modulatory balance of pro- and anti-inflammatory cytokines in pregnancy development that directly depends on the immunosuppressive action of IL-10.

From implantation until the end of a term pregnancy, the interaction between pro- and anti-inflammatory cytokines, of which IL-10 seems to be the key-cytokine, is fundamental to promote normal pregnancy outcomes. Recent findings of healthy pregnancy development show a global reduction of proinflammatory cytokines, such as TNF-*α*, IL-1*β*, and IL-6, and an increase of counterregulatory cytokines, such as IL-10. According to these studies, these alterations reinforce the role of immune modulation in the maintenance and development of normal pregnancy [6].

We sought to clarify the true role of cytokines in human pregnancy by conducting a review of the literature regarding the action of the cytokines IL-10 and TNF-*α* in pregnancy; we emphasized factors that are of interest to obstetrics.

## 2. Methods

The National Library of Medicine (Medline), *Literatura Latino-Americana e do Caribe em Ciências da Saúde* (LILACS), Scientific Electronic Library Online (Scielo), and PubMed were consulted for this review. National and international articles were located from the following key words: “cytokines,” “pregnancy,” “placenta,” “interleukin 10” and “tumor necrosis factor-alpha.” In some searches, these words were used in English. Initially, the articles were not limited to a specific time period; however, those that were published in the last 15 years were preferred. All authors evaluated the studies that were included in the review.

## 3. Discussion

### 3.1. IL-10 and Pregnancy

IL-10 is a particularly intriguing cytokine. In humans, it is characterized as pleiotropic and has dual immunologic functions. This dual function means that it is both stimulating and counterregulatory (immunosuppressive), which excludes it from the Th1/Th2 paradigm; however, it was originally described as a cytokine that displayed Th2 activity in rodents [6, 8, 9].

IL-10 is encoded on chromosome 1, and polymorphisms in the gene's promoter region can interfere with its regulation [32, 33]. Its receptor is composed of two subunits, IL-10R1 and IL-10R2, which are expressed in hematopoietic and nonhematopoietic cells, including the cytotrophoblast. The IL-10R1 receptor binds to the IL-10 protein; IL-10R2 is specific for initiating the signal transduction cascade that is necessary for its function. These signals involve the so-called Janus kinases (Jak) and transcription factors called signal transducers and transcription activators (STATs). After IL-10 binds to its receptor, the Jak enzymes phosphorylate tyrosine residues on the receptor, and they subsequently phosphorylate STAT in the cytoplasm. Sequentially, two STAT proteins bind to each other and dissociate from the receptor; the STAT dimers then migrate to the nucleus where they bind to DNA sequences in the promoter regions of IL10-responsive genes [32, 34].

IL-10 is the key cytokine at the beginning of pregnancy because it is involved in various important events, which include placental formation. IL-10 has a protective effect on the fetal-placental unit because it inhibits the secretion of inflammatory cytokines, such as IL-6, TNF-*α*, and IFN-*γ* [35]; jointly with IL-4 and IL-13, IL-10 seems to modulate trophoblastic invasion [12]. According to Thaxton and Sharma [32], IL-10 induces trophoblastic cells to produce vascular endothelial growth factor C (VEGF C) and the aquaporin (AQP1) system, which therefore stimulates placental angiogenesis. At the end of pregnancy, IL-10 production progressively decreases, and the concentrations of inflammatory cytokines predominate. According to Hanna et al. [36], this change in the cytokine profile is necessary to trigger spontaneous labor.

Other results have described decreased placental IL-10 production in the decidual region and the trophoblast in pregnancies that are complicated by intrauterine growth restriction [37], and this has been confirmed in experimental mice studies [38]. Conversely, higher IL-10 production was observed in normal pregnancies when compared with high-risk pregnancies; in the latter, the role of IL-10 in pregnancy maintenance and development is noteworthy [39]. A recent study on an experimental rat hypertension model revealed the normalization of blood pressure levels and endothelial function in association with IL-10 administration. According to those authors, the results reveal the importance of IL-10 action in conditions of exacerbated inflammatory response [40].

In summary, IL-10 is an important cytokine for pregnancy maintenance and development. During this specific period, its immunosuppressive action plays a key role in (i) regulating the balance of pro- and anti-inflammatory signs that orchestrate the adequate development of pregnancy and (ii) in placental growth and remodeling, which are also important for a favorable pregnancy outcome.

### 3.2. TNF-*α* and Pregnancy

TNF-*α* is an inflammatory cytokine that belongs to the subpopulation of Th1 lymphocytes, and it is encoded on chromosome 6. This cytokine regulates a number of cell functions, including cell proliferation, differentiation, and apoptosis. Macrophages are the primary producers of TNF-*α* and are also highly responsive to it [10].

TNF receptors are members of a large protein family, and they exist in almost every type of cell that is involved in immunologic and inflammatory responses. These receptors appear as trimers in the cytoplasmic membrane even after binding with TNF. When a cytokine binds to a type-I TNF receptor (TNF-RI), either gene expression or apoptosis may occur. The binding of the TRAAD adaptor protein (a death domain that is associated with the TNF receptor) occurs in the receptor's cytoplasmic domain, and this interaction is followed by the binding of two intermediate signaling factors, the TNF receptor-associated factor (TRAF) and the receptor interaction protein; this process promotes the gene expression of inflammatory mediators and antiapoptotic proteins (survival). To promote caspase activation and apoptosis induction, the TRAAD adaptor protein binds to the Fas-associated death domain (FAAD). Another action mechanism of such cytokines includes the direct binding of the cytoplasmic tails of other TNF receptor family members to FAAD, which leads to apoptosis or to TRAF and thus induces gene expression [34].

TNF-*α* increases with pregnancy development, and the primary production source of this cytokine appears to be the placenta [41]. Increased TNF-*α* can exacerbate insulin resistance, which is normal in pregnancy; this favors the development of GDM [14]. This characteristic is explained by the inhibition of the tyrosine-kinase activity of the insulin receptor in adipocytes and by the reduced phosphorylation and activation of the insulin receptor-1 substrate (IRS-1), which inhibits the insulin-signaling pathway [42]. Therefore, TNF-*α* regulation during pregnancy can prevent the deleterious effects of insulin resistance. According to some authors, such verification contradicts the classical concept that insulin resistance in pregnancy is exclusively induced by placental hormones such as progesterone, human chorionic gonadotropin, prolactin, and estradiol [43].

Various studies have evaluated the concentrations of circulating TNF-*α* in pregnant women with GDM; however, the results remain controversial. Some studies observed increased TNF-*α* in the blood of mothers who developed GDM [44–49]. However, other studies did not confirm such findings [50].

Significantly increased TNF-*α* production was observed in the placenta and the subcutaneous adipose tissue in women with GDM and noncontrolled hyperglycemia when compared with those with well-controlled GDM (i.e., normoglycemic). According to the authors, these observations suggest that the tissues of women with GDM increase the release of TNF-*α* in response to hyperglycemia. Because TNF-*α* is involved in the metabolic regulation of glucose, lipids, and insulin resistance, these data are consistent with the hypothesis that TNF-*α* is involved in GDM development [14, 51].

Other effects of TNF-*α* have been described in the newborns of diabetic and nondiabetic mothers and in other high-risk pregnancies. TNF-*α* decreases in the macrosomic offspring of mothers with GDM [45], and contrarily, it increases in the placentas of pregnancies that are complicated by fetal growth restriction [52]. Increased TNF-*α* concentrations were observed in pregnant women with preeclampsia (hypertension with proteinuria) when compared with those with gestational hypertension (hypertension without proteinuria). This result suggests that TNF-*α* can be used as a marker of severity in pregnancy hypertensive syndromes [13].

In summary, TNF-*α* is related to obesity, glucose intolerance, type-2 diabetes mellitus, and GDM, and it is positively correlated with body mass index (BMI) [14, 44, 48, 53]. In pregnancy, TNF-*α* production in maternal adipose tissue is enhanced by the placental production of TNF-*α*, which makes it an important factor in the pathogenesis of insulin resistance and GDM. This challenges the traditional theory that reproductive hormones alone reduce insulin sensitivity during pregnancy [14, 43, 49, 54]. Although this has not been completely defined, TNF-*α* seems to be the marker of “demetabolism” and maternal glycemic control in pregnancies that are complicated by type-2 diabetes mellitus and GDM.

## 4. Conclusion and Future Prospects

The literature describes various functions of IL-10 and of TNF-*α* in normal pregnancy. The actions of these cytokines seem to be antagonistic and dependent on the balance between them, which is orchestrated by the specific immunosuppressive action of IL-10. TNF-*α* has a characteristic inflammatory action, and it is an additional diabetogenic factor in pregnancy. The loss of the control of the production of these cytokines, with increase of TNF-*α*, is related to the risk for developing obstetric complications, particularly recurrent fetal loss, GDM, hypertensive syndromes, and fetal growth restriction. However, these results remain controversial and are not completely reproducible. These issues are attributed to the heterogeneity of the studies, which are especially related to their source (*in vivo* compared with *in vitro*), sample size (which is usually insufficient), evaluation methods (immunohistochemistry or molecular biology tools), and the multiplicity of factors and conditions that influence cytokine production. These issues are fundamental and must be considered in future investigations to produce more consistent results that can benefit the field of obstetrics.
